# Religious affiliation and oral health-related quality of life: a cross-sectional study based on a nationally representative survey in Germany

**DOI:** 10.1186/s12903-023-03265-8

**Published:** 2023-08-23

**Authors:** Nina Moszka, Ghazal Aarabi, Berit Lieske, Hans-Helmut König, Benedikt Kretzler, Larissa Zwar, André Hajek

**Affiliations:** 1 Present Address: Department of Periodontics, Preventive and Restorative Dentistry, Center for Dental and Oral Medicine, University Medical Center Hamburg Eppendorf, Hamburg, Germany; 2 Department of Health Economics and Health Services Research, University Medical Center Hamburg-Eppendorf, Hamburg Center for Health Economics, Hamburg, Germany

**Keywords:** Oral health, Oral health-related quality of life, Religious affiliation, Religiosity, Religious denomination

## Abstract

**Background:**

Studies have shown an association between a person’s religiosity, and physical as well as psychological, health status. However, results differ between certain religious affiliations. While good oral health is important for our overall health and wellbeing, research on religious affiliation and oral health status, specifically oral health-related quality of life (OHRQoL), is lacking. Thus, our aim was to investigate the association between religious affiliation and OHRQoL.

**Methods:**

A nationally representative online survey (n = 3,075 individuals) was conducted in August/September 2021. The mean age was 44.5 years (SD: 14.8 years, 18 to 70 years) and 51.1% of the individuals were female. OHRQoL was measured using the Oral Health Impact Profile (OHIP-G5). Religious affiliation served as key explanatory variable. Several covariates were included in regression analyses.

**Results:**

Regressions revealed that compared to individuals with no religious affiliation, individuals belonging to Christianity had poorer OHRQoL (β = 0.31, p < 0.01), individuals belonging to Islam had poorer OHRQoL (β = 2.62, p < 0.01) and individuals belonging to another religious affiliation also had poorer OHRQoL (β = 1.89, p < 0.01).

**Conclusion:**

Our study demonstrated an association between religious affiliation and OHRQoL. Individuals with specific religious affiliations should be addressed to avoid low OHRQoL.

**Supplementary Information:**

The online version contains supplementary material available at 10.1186/s12903-023-03265-8.

## Introduction

The relationship between religion and health outcomes has been of longstanding interest in epidemiological research [1]. Religion is a multidimensional construct, generally associated with specific beliefs and practices. In order to assess religiosity, which refers to a person’s religious orientation, conviction and involvement, different aspects may be considered: religious affiliation, attendance at religious services, religious salience (or intrinsic religiosity), personal importance and commitment, and private religious practices [2–4]. Religious affiliation is defined as “the self-identified association of a person with a religion, denomination or sub-denominational religious group” [5].

### Religiosity and health outcomes

Greater religious involvement, particularly greater personal commitment or religious salience and attendance at religious services, has been associated with higher levels of life satisfaction [6–8], health-related quality of life [9–11], better self-rated health [1, 6, 7, 9, 11–15], and a reduction in all-cause mortality risk [4, 16–20]. Furthermore, studies demonstrate a protective effect of religiosity on mental health outcomes [21, 22].

Good oral health is important for the maintenance of people’s overall health and well-being [23]. Overall, literature on the relationship between religion and oral health is scarce, with only a few studies reporting on the association between religiosity and oral health [24–30]. For example, the frequency of attending religious services has been reported to be positively associated with preventive dental checkups [24, 29], better self-rated oral health [30] and better oral health outcomes [27, 28]. Furthermore, a study with 1,134 12-year-old Brazilian school children demonstrated that family religiosity, measured by attendance at religious services and private religious practices, was positively associated with school children’s oral health-related quality of life (OHRQoL) [25].

### Religious affiliation and health

Despite the increasing interest in the role of religion and health, differences between religious groups (and people with no religious affiliation) are less frequently addressed. Studies report differing health outcomes [31–37] across various religious groups; and according to whether individuals belong to a minority or majority religious group in their respective country. In a study on religious affiliation and COVID-19-related mortality, those with Jewish affiliation demonstrated higher risk of death in comparison to all other groups (Christians, Muslims, Hindus, Sikhs, Buddhists, and no religious affiliation) [38]. Mortality differences by religious groups were also reported by a cross-sectional study with a cohort of Black Protestants, Evangelic Protestants, Catholics, Mainline Protestants, Jews, and individuals with no affiliation [36], with. Mainline Protestants demonstrating a mortality advantage relative to the others [36]. Differences in illness-related behavior and utilization of health services have also been reported between different religious affiliations [26, 35, 39]. Variations in oral health status, specifically OHRQoL, between religious affiliations, however, have rarely been examined.

### Study aim

In light of the limited knowledge on the association between religiosity and oral health, the aim of the current cross-sectional study is to investigate the association between religious affiliation and OHRQoL. For this purpose, we used data from a nationally representative survey among the general adult population in Germany.

## Methods

### Sample

Our study meets the STROBE guidelines for the reporting of cross-sectional studies (please see the Supplementary File 1).

We based our findings on data from a nationally representative survey. A total of n = 3,075 individuals aged 18 to 70 and residing in Germany were included in the study. The data was gathered between the end of August and the beginning of September of 2021. Respondi, a market research firm, recruited participants through its own online panel. In terms of age bracket, sex and federal state, recruitment was representative (quota-based). About 14,000 individuals were contacted in total. Because this was an online sample, no sample selection bias could be calculated. Missing values were not present in the main regression model.

All participants included in this study provided informed consent. Approval for the study was provided by the Local Psychological Ethics Committee of the Center for Psychosocial Medicine of the University Medical Center Hamburg-Eppendorf (number: LPEK-0356). Our study is in accordance with the ethical standards laid down in the 1964 Declaration of Helsinki and its later amendments.

### Outcome: oral health-related quality of life

To measure OHRQoL, the established Oral Health Impact Profile (OHIP-G5) [40], which consists of five items, was used. It is divided into four categories [41]: [1] oral function, [2] orofacial pain, [3] appearance, and [4] psychosocial impact. The items referring to “difficulty chewing foods” and “less flavor in food” correspond to the category oral function. The item referring to “painful aching” correspond to orofacial pain. The item referring to “uncomfortable about appearance” pertains to appearance. Lastly, the item referring to “difficulty doing your usual jobs” refers to psychosocial impact. Responses were made on a Likert-type scale (0 = never, 1 = hardly ever, 2 = occasionally, 3 = fairly often, 4 = very often). Thus, based on all five items, the total score ranges between 0 and 20. It should be noted that higher scores reflect lower OHRQoL. In our study, Cronbach’s alpha was 0.85. Former research has also shown good to very good psychometric properties of the OHIP-G5 [40].

### Independent variables

Our independent variable of interest was religious affiliation. In accordance with other large cohort studies (e.g., German Ageing Survey), individuals were asked: “Which denomination do you belong to?” (Not belonging to any denomination; Christianity; Judaism; Islam; Buddhism; Hinduism; Other). Due to the number of cases, we categorized the participants into these four categories: No denomination, Christianity, Islam, and other.

In our regression analyses, several covariates were included: age, sex (men; women; diverse), marital status (distinguishing between: married, living together with spouse; married, not living together with spouse; widowed; single; divorced), employment status (full-time employed; retired; other), education (upper secondary school; qualification for applied upper secondary school; polytechnic secondary school; intermediate secondary school; lower secondary school; currently in school training/education; without school-leaving qualification), alcohol intake (daily; several times per week; once a week; 1–3 times per month; less often; never), smoking status (never smoker; no, not anymore; yes, sometimes; yes, daily), chronic diseases (absence of chronic diseases; presence of at least one chronic disease) and self-rated health (from 1 = very bad to 5 = very good).

### Statistical analysis

Sample characteristics are displayed stratified by religious affiliation. Some exemplary effect sizes (Cohen’s d [42]) were calculated for religious affiliation and OHRQoL. In a further step, unadjusted and adjusted linear regressions were performed to investigate the association between religious affiliation and OHRQoL (also for the four categories (five items) as outcomes). The overall score served as primary outcome and the single items of the OHIP-G5 served as secondary outcomes.

In a sensitivity analysis, the main model was extended by adding migration background and income category as covariates. Migration background was quantified based on self-reports (no; yes). An explanation was added as follows: “A person has a migration background if he or she or at least one parent was not born with German citizenship”. Moreover, 13 income categories (based on the household net income) were used (under EUR 500, 500 EUR to lower than EUR 1000, EUR 1000 to lower than EUR 1500, EUR 1500 to lower than EUR 2000, EUR 2000 to lower than EUR 2500, EUR 2500 to lower than EUR 3000, EUR 3000 to lower than EUR 3500, EUR 3500 to lower than EUR 4000, EUR 4000 to lower than EUR 4500, EUR 4500 to lower than EUR 5000, EUR 5000 to lower than EUR 6000, EUR 6000 to lower than EUR 8000, EUR 8000 or more).

The significance level was set at p < 0.05. For the statistical analyses, Stata 16.1 (Stata Corp., College Station, Texas) was used.

## Results

### Sample characteristics stratified by religious affiliation and effect sizes

In the total sample, the mean age was 44.5 years (18 to 70 years; Standard Deviation (SD): 14.8 years), with 51.1% being female. Sample characteristics stratified by religious affiliation are shown in Table 1.

**Table 1:** Sample characteristics stratified by religious affiliation (n = 3,075)

	Religious affiliation			
Variables	No denomination	Christianity	Islam	Other
	N = 1,401	N = 1,575	N = 49	N = 50
Oral health-related quality of life (OHIP-G5; ranging from 0 to 20, with higher values reflecting lower oral health-related quality of life)	2.0 (3.1)	2.2 (3.4)	4.7 (5.3)	3.9 (4.5)
Oral function: difficulty chewing foods (from 0 = never to 4 = very often)	0.5 (0.9)	0.5 (0.9)	1.0 (1.2)	0.9 (1.1)
Oral function: less flavor in food (from 0 = never to 4 = very often)	0.3 (0.7)	0.4 (0.7)	1.1 (1.2)	0.9 (1.2)
Orofacial pain: painful aching (from 0 = never to 4 = very often)	0.4 (0.8)	0.5 (0.9)	0.8 (1.2)	0.7 (0.9)
Appearance: Uncomfortable about appearance (from 0 = never to 4 = very often)	0.5 (1.0)	0.6 (1.0)	1.0 (1.1)	1.0 (1.3)
Psychosocial impact: Difficulty doing your usual jobs (from 0 = never to 4 = very often)	0.2 (0.6)	0.3 (0.7)	0.9 (1.2)	0.5 (0.9)
Sex				
Men	734 (52.4%)	719 (45.7%)	24 (49.0%)	25 (50.0%)
Women	665 (47.5%)	855 (54.3%)	25 (51.0%)	25 (50.0%)
Diverse	2 (0.1%)	1 (0.1%)	0 (0.0%)	0 (0.0%)
Age	45.5 (14.5)	44.1 (14.9)	31.7 (10.7)	44.6 (16.4)
Marital status				
Single / Divorced / Widowed / Married, not living together with spouse	593 (42.3%)	664 (42.2%)	26 (53.1%)	30 (60.0%)
Married, living together with spouse	808 (57.7%)	911 (57.8%)	23 (46.9%)	20 (40.0%)
Highest educational degree				
upper secondary school	599 (42.8%)	683 (43.4%)	23 (46.9%)	21 (42.0%)
qualification for applied upper secondary school	152 (10.8%)	165 (10.5%)	7 (14.3%)	4 (8.0%)
polytechnic Secondary School	131 (9.4%)	31 (2.0%)	1 (2.0%)	5 (10.0%)
intermediate Secondary School	378 (27.0%)	485 (30.8%)	13 (26.5%)	12 (24.0%)
lower Secondary School	133 (9.5%)	201 (12.8%)	5 (10.2%)	8 (16.0%)
currently in school training/education	4 (0.3%)	5 (0.3%)	0 (0.0%)	0 (0.0%)
without school-leaving qualification	4 (0.3%)	5 (0.3%)	0 (0.0%)	0 (0.0%)
Employment status				
Full-time employed	724 (51.7%)	699 (44.4%)	21 (42.9%)	14 (28.0%)
Retired	235 (16.8%)	252 (16.0%)	0 (0.0%)	12 (24.0%)
Other	442 (31.5%)	624 (39.6%)	28 (57.1%)	24 (48.0%)
Smoking status				
Yes, daily	346 (24.7%)	349 (22.2%)	13 (26.5%)	8 (16.0%)
Yes, sometimes	130 (9.3%)	108 (6.9%)	9 (18.4%)	4 (8.0%)
No, not anymore	405 (28.9%)	414 (26.3%)	8 (16.3%)	16 (32.0%)
No, never	520 (37.1%)	704 (44.7%)	19 (38.8%)	22 (44.0%)
Alcohol intake				
Daily	96 (6.9%)	84 (5.3%)	3 (6.1%)	3 (6.0%)
Several times per week	281 (20.1%)	273 (17.3%)	5 (10.2%)	5 (10.0%)
Once a week	239 (17.1%)	243 (15.4%)	10 (20.4%)	3 (6.0%)
1–3 times per month	250 (17.8%)	269 (17.1%)	6 (12.2%)	7 (14.0%)
Less often	293 (20.9%)	396 (25.1%)	9 (18.4%)	17 (34.0%)
Never	242 (17.3%)	310 (19.7%)	16 (32.7%)	15 (30.0%)
Chronic diseases				
Absence of chronic diseases	814 (58.1%)	888 (56.4%)	39 (79.6%)	24 (48.0%)
Presence of at least one chronic disease	587 (41.9%)	687 (43.6%)	10 (20.4%)	26 (52.0%)
Self-rated health (1 = very bad to 5 = very good)	3.6 (0.9)	3.6 (0.9)	3.9 (0.8)	3.5 (0.9)

Individuals without religious affiliation had a mean OHRQoL score of 2.0 (SD: 3.1), individuals belonging to Christianity had a mean OHRQoL score of 2.2 (SD: 3.4), individuals belonging to Islam had a mean OHRQoL score of 4.7 (SD: 5.3), and individuals belonging to another religious affiliation had a mean OHRQoL score of 3.9 (SD: 4.5). Further details (e.g., for the dimensions) are provided in Table 1.

With respect to effect sizes (Cohen’s d); individuals without religious affiliation had a better OHRQoL (Cohen’s d=-0.07) compared to individuals belonging to Christianity for example. Such an effect size can be considered as negligible. Another example: Individuals without religious affiliation had a better OHRQoL (Cohen’s d=-0.86) compared to individuals belonging to Islam. This difference can be considered as large.

### Regression analysis

Results of multiple linear regressions are given in Table 2 (unadjusted) and in Table 3 (adjusted). Due to the higher meaningfulness of the adjusted results, in this section we focus on reporting the results of the adjusted regressions (Table 3). However, the unadjusted results can be found in detail in Table 2.

**Table 2:** Religious affiliation and oral health-related quality of life. Findings of linear regressions (unadjusted)

Independent variables	Oral health-related quality of life	Oral function: difficulty chewing foods	Oral function: less flavor in food	Orofacial pain: painful aching	Appearance: Uncomfortable about appearance	Psychosocial impact: Difficulty doing your usual jobs
Religious affiliation: - Christianity (Ref.: No denomination)	0.24*	0.03	0.03	0.06+	0.07*	0.04+
	(0.12)	(0.03)	(0.03)	(0.03)	(0.04)	(0.02)
- Islam	2.73***	0.51**	0.72***	0.40*	0.44**	0.66***
	(0.76)	(0.18)	(0.18)	(0.17)	(0.16)	(0.18)
- Other	1.93**	0.37*	0.60***	0.24+	0.42*	0.30*
	(0.63)	(0.15)	(0.17)	(0.13)	(0.18)	(0.13)
Observations	3,075	3,075	3,075	3,075	3,075	3,075
R²	0.02	0.01	0.02	0.00	0.01	0.02

Unstandardized beta-coefficients are displayed; robust standard errors (SE) in parentheses; *** p < 0.001, ** p < 0.01, * p < 0.05, + p < 0.10

**Table 3:** Religious affiliation and oral health-related quality of life. Findings of multiple linear regressions

Independent variables	Oral health-related quality of life	Oral function: difficulty chewing foods	Oral function: less flavor in food	Orofacial pain: painful aching	Appearance: Uncomfortable about appearance	Psychosocial impact: Difficulty doing your usual jobs
Religious affiliation: - Christianity (Ref.: No denomination)	0.31** (0.12)	0.05+	0.06*	0.06*	0.08*	0.06*
		(0.03)	(0.03)	(0.03)	(0.04)	(0.03)
- Islam	2.62** (0.75)	0.57**	0.71***	0.35*	0.38*	0.61***
		(0.18)	(0.17)	(0.17)	(0.16)	(0.17)
- Other	1.89** (0.62)	0.37*	0.59***	0.22+	0.40*	0.31*
		(0.15)	(0.17)	(0.13)	(0.18)	(0.13)
Covariates	✓	✓	✓	✓	✓	✓
Observations	3,075	3,075	3,075	3,075	3,075	3,075
R²	0.10	0.07	0.08	0.06	0.08	0.07

Unstandardized beta-coefficients are displayed; robust standard errors (SE) in parentheses; *** p < 0.001, ** p < 0.01, * p < 0.05, + p < 0.10; Covariates include sex, age, family status, education, employment status, smoking status, alcohol intake, presence of chronic diseases and self-rated health

In Table 3, the R² value was 0.10 (with OHIP-G5 score as outcome). Regressions revealed that compared to individuals without religious affiliation, individuals belonging to Christianity had higher OHIP-G5 scores (β = 0.31, p < 0.01). It is worth repeating that higher OHIP-G5 scores reflect a lower OHRQoL. Moreover, compared to individuals without religious affiliation, individuals belonging to Islam had higher OHIP-G5 scores (β = 2.62, p < 0.01) and individuals belonging to another religious affiliation also had higher OHIP-G5 scores (β = 1.89, p < 0.01). When the four categories ([1] oral function, [2] orofacial pain, [3] appearance, and [4] psychosocial impact) served as outcome measures, comparable findings were observed in terms of significance. Please see Table 3 for further details.

In a sensitivity analysis, the main model was extended by adding migration background and income category (please see the Supplementary File 2). However, in terms of significance and effect size, the association between religious affiliation and the outcomes remained very similar.

## Discussion

### Main findings

Using data from a large representative survey, our aim was to investigate the association between religious affiliation and OHRQoL. Regressions revealed that compared to individuals without religious affiliation, individuals belonging to Christianity, Islam or another religious affiliation had poorer OHRQoL. Our study adds to research on religion and oral health, with this study being the first to examine the association between religious affiliation and OHRQoL in adults.

### Previous research and possible explanations

The extent to which one belongs or identifies, and is involved, with a particular religion or religious group may inadvertently define a lifestyle that affects one’s health. For example, the majority of religious institutions prohibit or discourage behaviors that are damaging to one’s health (e.g. alcohol consumption) and instead endorse health-promoting behaviors in accordance with their religious beliefs [19, 39, 43–48]. Overall, religious activities, organizational activities (e.g. going to church) or private practices (e.g. private prayer) and religious commitment have been described as an important feature of bonding, coping with stress or illness and encouraging healthy behavior, which in turn might explain better mental and physical health outcomes, as well as the inverse association between religiosity and all-cause mortality reported by some studies [11, 14–18, 49–51]. However, some studies also report on the negative impacts of religiosity on health outcomes [7, 52–57]. Religious struggle (e.g. feelings of abandonment by God or of being punished due to a lack of religious devotion, questioning of beliefs, or conflict with religious others) may contribute to illness and be a predictor of increased risk of death [53] and negative mental health outcomes [54, 55, 58]. In a study among Greek Orthodox Christians, higher frequency of private religious practices was positively associated with levels of anxiety [7].

In contrast to many previous studies that have reported a positive association between aspects of religiosity and health-related quality of life, we found that OHRQoL was best in non-affiliated individuals. It has been theorized that people with religious affiliation might be less likely to seek health care, possibly because of a tendency to defer control over one’s health to God or another higher power, or by seeing religion as intervention in place of treatment, which might negatively affect utilization patterns [52, 56, 59]. Non-utilization patterns have been associated with poorer oral health outcomes, which affect OHRQoL [60–62]. According to a study by Christy and colleagues [63] on psychosocial variables that predict being at risk for low health literacy, the authors found that greater reliance on religious beliefs for medical decision-making was significantly associated with being at risk for low health literacy. Lower health literacy is not just associated with poorer oral hygiene behaviors and oral health outcomes [64–66], but also lower OHRQoL [67, 68]. This might be a possible explanation as to why study participants with no religious affiliation had higher OHRQoL; however, further longitudinal data would be helpful in testing this hypothesis.

Previous research has shown that health outcomes and health behaviors differ depending on the religion to which the participant belongs [32, 34, 38, 69]. We found one other study that compared the OHRQoL between participants associated with different religious affiliations [70]. In their cross-sectional study among Ethiopian special needs students, the authors found that students affiliated with the Orthodox religion, one of the Christian churches in Ethiopia, had better OHRQoL compared to those affiliated with all other religions (Catholic, Muslim and Protestant). Similar to our results, Muslim affiliated students scored highest in the assessment, indicating lower OHRQoL. The authors, however, did not include people with no religious affiliation in their study.

As 6.5% of the total population, Muslims represent a minority religious group in Germany [71]. Empirical research shows that Muslims in particular often face discrimination due to their religious background [72]. Health disadvantages among minority groups were also reported elsewhere [34]. When it comes to oral health, research shows that Christian affiliated individuals are more likely to use oral health care facilities than people with other affiliations [26] and report better oral health behavior than Muslims [33]. The Muslim population in Germany has almost exclusively of a migration background [71]. Recent studies showed that people with a migration background in Germany demonstrated poorer oral health literacy levels and oral health status than people without a migration background [73, 74]. Another possible explanation for the differences in OHRQoL in our study might be the lack of knowledge and understanding of the specific needs of Muslim patients among Western healthcare providers [75]. Religious groups not only share beliefs, but most likely also ethnic, cultural and socioeconomic similarities, that should also be considered. This leads to the clinical implication that cross-cultural knowledge, which takes religious dimensions into account, is necessary to adopt culturally acceptable behaviors, strengthen patient-provider relationships and optimize therapeutic outcomes [75].

Overall, it is important to note that all religions are extremely diverse and comprised of an extremely heterogeneous group. Additionally, being affiliated with a religion does not necessarily determine a person’s personal religious conviction or that one must actively perform religious practices. For example, using data from the General Social Survey-National Death Index, Kim and colleagues found that while affiliation with a religion corresponded to the strength of religiosity for some religious groups, it did not for others [32]. Data on private or personal aspects of religiosity (e.g. religious importance or religious salience) as well as public aspects (e.g. attendance at religious services) was not captured by our study. This may account for the differing findings to those of previous research.

### Strengths and limitations

We used data from a survey which reflects the distribution of age, sex and federal state in the general adult population in Germany. An established and valid tool with sufficient discriminative and evaluative psychometric properties was used to quantify our outcome measure (OHRQoL) [40]. A short version of the OHIP was also developed in Spanish and found to be a valid and reliable instrument [76]. Moreover, in our regression analyses, we adjusted for various covariates.

Due to the rising number of migrants and asylum seekers from countries with a large Muslim population, such as Turkey, Syria or Afghanistan, the number of Islam affiliated people in Germany is rising. However, because of their migration status, German language skills among this subpopulation may be lacking. The result of a language screening test in Hamburg showed, for example, that 31% of the Turkish migrants of the so-called first generation (“guest workers”) have no German language skills [77]. The OHIP-G5 questionnaire was only available in the German language for the survey participants. This means that individuals with certain religious affiliations may be underrepresented in our study.

The only aspect of religion investigated is religious affiliation. Other aspects relevant to research in this field, such as frequency of attending religious services, personal involvement and belief as well as religious practices, are not taken into account. Lastly, our current study is a cross-sectional one – with the known limitation regarding causality.

## Conclusions

Our study demonstrated an association between religious affiliation and OHRQoL. We found that individuals affiliated with one of the religious denominations (Christianity, Islam, Other) had poorer OHRQoL than those without religious affiliation. Thus, individuals with certain religious affiliations should be specifically addressed to avoid low OHRQoL. However, due to the heterogeneity of religious groups, and as the measurability and operationalization of ‘religion’ remains a problem, additional aspects of religiosity should be considered. Overall, further research on health and religious affiliation is warranted. We conclude that a more culturally sensitive approach to oral health promotion, which takes account of religious dimensions of health behaviour, must be adopted.

### Electronic supplementary material

Below is the link to the electronic supplementary material.

Supplementary Material 1

Supplementary Material 2

## Data Availability

The datasets generated and/or analyzed during the current study are not publicly available due to legal restrictions but are available from the corresponding author on reasonable request.

## Abbreviations

e.g.: exempli gratia (lat.)
etc.: et cetera (lat.)
OHRQoL: Oral Health-Related Quality of Life
OHIP: Oral Health Impact Profile
SD: Standard Deviation
