# Supplementary material for: Religious affiliation and oral health-related quality of life: a cross-sectional study based on a nationally representative survey in Germany
Source: BMC Oral Health. 2023 Aug 23;23:586. doi: 10.1186/s12903-023-03265-8 (PMC10463515; doi:10.1186/s12903-023-03265-8)
Supplement: Supplementary file 2 — Supplementary Material 2 [file 12903_2023_3265_MOESM2_ESM.docx]

Supplementary File 2. Religious affiliation and oral health-related quality of life. Findings of multiple linear regressions (extended model additionally adjusting for migration background and income category).

| Independent variables | Oral health-related quality of life | Oral function: difficulty chewing foods | Oral function: less flavor in food | Orofacial pain: painful aching | Appearance: Uncomfortable about appearance | Psychosocial impact: Difficulty doing your usual jobs |
| --- | --- | --- | --- | --- | --- | --- |
|  |  |  |  |  |  |  |
| Religious affiliation: - Christianity (Ref.: No denomination) | 0.31* | 0.06 | 0.05+ | 0.06+ | 0.08* | 0.06* |
|  | (0.13) | (0.03) | (0.03) | (0.03) | (0.04) | (0.03) |
| - Islam | 2.86** | 0.64** | 0.77*** | 0.41* | 0.39* | 0.65** |
|  | (0.88) | (0.21) | (0.20) | (0.20) | (0.19) | (0.21) |
| - Other | 1.99** | 0.40** | 0.64*** | 0.25+ | 0.41* | 0.29* |
|  | (0.66) | (0.16) | (0.18) | (0.14) | (0.19) | (0.14) |
| Covariates | ✓ | ✓ | ✓ | ✓ | ✓ | ✓ |
|  |  |  |  |  |  |  |
| Observations | 2,761 | 2,761 | 2,761 | 2,761 | 2,761 | 2,761 |
| R² | 0.12 | 0.08 | 0.09 | 0.08 | 0.10 | 0.09 |

Unstandardized beta-coefficients are displayed; robust standard errors (SE) in parentheses; *** p<0.001, ** p<0.01, * p<0.05, + p<0.10; Covariates include sex, age, family status, education, employment status, smoking status, alcohol intake, presence of chronic diseases and self-rated health – as well as migration background and income category.
